# Portable HEPA filter air cleaner use during pregnancy and children’s behavior problem scores: a secondary analysis of the UGAAR randomized controlled trial

**DOI:** 10.1186/s12940-021-00763-6

**Published:** 2021-07-05

**Authors:** Undarmaa Enkhbat, Enkhjargal Gombojav, Chimeglkham Banzrai, Sarangerel Batsukh, Buyantushig Boldbaatar, Enkhtuul Enkhtuya, Chimedsuren Ochir, David C. Bellinger, Bruce P. Lanphear, Lawrence C. McCandless, Ryan W. Allen

**Affiliations:** 1 Faculty of Health Sciences, Simon Fraser University, Burnaby, BC Canada; 2 School of Public Health, Mongolian National University of Medical Sciences, Ulaanbaatar, Mongolia; 3 Institute of Medical Sciences, Mongolian National University of Medical Sciences, Ulaanbaatar, Mongolia; 4 School of Graduate Studies, Mongolian National University of Medical Sciences, Ulaanbaatar, Mongolia; 5 Harvard Medical School, Boston Children’s Hospital, Boston, MA USA

**Keywords:** RCT, Intervention, Prenatal, DOHaD, Programming

## Abstract

**Background:**

Developmental exposure to particulate matter (PM) air pollution may impair children’s behaviors. Our objectives were to quantify the impact of reducing indoor PM using portable HEPA filter air cleaners during pregnancy on behavioral problems in children and to assess associations between indoor fine PM (PM_2.5_) concentrations during pregnancy and children’s behavior.

**Methods:**

This is a secondary analysis of a single-blind parallel-group randomized controlled trial in which we randomly assigned 540 non-smoking pregnant women to receive 1 or 2 HEPA filter air cleaners or no air cleaners. We administered the Behavior Assessment System for Children (BASC-3) to caregivers when children were a mean age of 23 months, and again at a mean age of 48 months. Primary outcomes were the four BASC-3 composite scales: externalizing problems, internalizing problems, adaptive skills, and the behavioral symptoms index. We imputed missing data using multiple imputation with chained equations. The primary analysis was by intention-to-treat. In a secondary analysis, we evaluated associations between BASC-3 composite indices and modeled trimester-specific PM_2.5_ concentrations inside residences.

**Results:**

We enrolled participants at a median of 11 weeks gestation. After excluding miscarriages, still births and neonatal deaths, our analysis included 478 children (233 control and 245 intervention). We observed no differences in the mean BASC-3 scores between treatment groups. An interquartile increase (20.1 µg/m^3^) in first trimester PM_2.5_ concentration was associated with higher externalizing problem scores (2.4 units, 95% CI: 0.7, 4.1), higher internalizing problem scores (2.4 units, 95% CI: 0.7, 4.0), lower adaptive skills scores (-1.5 units, 95% CI: -3.0, 0.0), and higher behavior symptoms index scores (2.3 units, 95% CI: 0.7, 3.9). Third trimester PM_2.5_ concentrations were also associated with some behavioral indices at age 4, but effect estimates were smaller. No significant associations were observed with PM_2.5_ concentrations during the second trimester or for any of the BASC indices when children were 2 years old.

**Conclusion:**

We found no benefit of reducing indoor particulate air pollution during pregnancy on parent-reported behaviors in children. Associations between indoor PM_2.5_ concentrations in the first trimester and behavioral scores among 4-year old children suggest that it may be necessary to intervene early in pregnancy to protect children, but these exploratory findings should be interpreted cautiously.

**Trial registration:**

ClinicalTrials.gov: NCT01741051

**Supplementary Information:**

The online version contains supplementary material available at 10.1186/s12940-021-00763-6.

## Introduction

Exposure to fine particulate matter air pollution (PM_2.5_) during pregnancy is linked to impaired fetal growth [1, 2], which may in turn cause developmental programming that adversely affects health in childhood and beyond [3]. Evidence from animal and epidemiologic studies suggests that prenatal exposure to air pollution may adversely affect brain development [4–6]. Results from birth cohort studies of prenatal exposure and children’s behaviors have varied [7–14].

Given the speed and complexity of brain development, the impact of an exposure may depend on timing [11, 15, 16]. Two studies of PM exposure and behavior problems in childhood have evaluated the impacts of exposure during specific stages of pregnancy and suggested that exposures in the first and third trimesters may be most important [7, 11].

Investigators have not definitively identified the biological mechanism(s) through which air pollution exposure in pregnancy may impact brain development, but such a link is biologically plausible. Systematic inflammation may play a role [17]. Animal experiments have suggested that prenatal exposure to airborne particles induces inflammation in mothers followed by changes in brain morphology in the offspring [4, 18].

Portable high efficiency particulate air (HEPA) filter air cleaners (“HEPA cleaners”) reduce indoor PM_2.5_ concentrations by 29–82% [19, 20]. Because outdoor air pollution infiltrates into buildings, indoor air contains pollution emitted from both outdoor and indoor sources. As a result, a substantial portion of the health impacts from outdoor pollution sources stem from exposure that occurs indoors. For example, indoor exposures account for 61% and 81% of the deaths attributed to outdoor-generated PM_2.5_ in the US and China, respectively [21, 22]. Thus, reducing particle concentrations indoors may mitigate the health impacts of outdoor pollution sources.

To date, no studies have evaluated the impact of reducing PM_2.5_ during pregnancy on behavioral problems in childhood. The objective of this randomized controlled trial (RCT) was to quantify the impact of reducing PM_2.5_ using HEPA cleaners during pregnancy on behavioral problems in children at 2 and 4 years of age. In addition, we sought to explore associations between indoor PM_2.5_ during different periods of pregnancy and behavior in these children.

## Materials and methods

The Ulaanbaatar Gestation and Air Pollution Research (UGAAR) study is a single-blind parallel-group RCT. The study was originally designed to estimate the effect of PM exposure reductions from portable HEPA cleaner use during pregnancy on fetal growth (ClinicalTrials.gov: NCT01741051). The study was later extended to include observations of early childhood development. We conducted the trial in Ulaanbaatar, Mongolia’s polluted capital city, where the primary source of PM_2.5_ is coal combustion in home heating stoves [23–26]. The UGAAR study protocol was approved by the Simon Fraser University Office of Research Ethics and the Mongolian Ministry of Heath Medical Ethics Approval Committee.

### Participants

We recruited participants at two perinatal health clinics in the centrally located Sukhbaatar district of Ulaanbaatar. We enrolled a total of 540 non-smoking women who met the following criteria: ≥ 18 years of age, ≤ 18 weeks into a single gestation pregnancy, non-smoker, living in an apartment, not using portable air cleaner(s) at enrollment, and planning to give birth in a medical facility in Ulaanbaatar.

All participants provided written informed consent prior to data collection. We compensated participants up to 65,000 Mongolian tugriks (approximately $30 USD) during prenatal follow-up and up to 260,000 tugriks (approximately $100 USD) during the 4 years of post-natal follow-up. Compensation was pro-rated based on the specific activities that participants completed.

### Randomization and blinding

Participants were randomly assigned to the control or intervention group on a 1:1 ratio using sealed opaque envelopes containing randomly generated cards indicating “filter” or “control”. In this single-blind trial participants were not blinded to intervention status but staff responsible for outcome assessment were blinded. After a woman provided consent, a study coordinator drew an envelope in sequential order, opened it, and informed the participant of her allocation. The envelope was then discarded and a new one was opened when the next participant was enrolled.

### Intervention

The intervention group received one or two HEPA cleaners (Coway AP-1009CH) depending on the size of the home. In smaller apartments (< 40 m^2^) we placed an air cleaner in the main living area of the home; in larger apartments (≥ 40 m^2^), we placed a second air cleaner in the participant’s bedroom. We deployed the HEPA cleaners in intervention homes shortly after enrollment into the study. The control group received no HEPA cleaners. We did not replace the HEPA filter(s) during the study and we collected the HEPA cleaner(s) after pregnancy ended.

### Prenatal data collection

Participants visited our study office shortly after enrollment, between 5 and 19 weeks gestation, and again at 24–37 weeks gestation [27]. At both visits we administered questionnaires on demographics, lifestyle, housing, and health. Participants completed the 4-question perceived stress scale (PSS4) as part of both prenatal questionnaires [28]. During the second visit, we also collected a venous whole blood sample, which was analyzed within 6 weeks of collection for lead, mercury, and cadmium concentrations using quadrupole-based inductively coupled plasma-mass spectrometry (ICP-MS), with matrix-matched calibration [27, 29].

We measured indoor PM_2.5_ in participants’ apartments over 7 days at a median of 11 weeks (shortly after enrollment and air cleaner deployment) and again at a median of 30 weeks using Dylos DC 1700 laser particle counters. As described elsewhere, these measurements were used to develop a blended multiple linear regression / random forest regression prediction model that provides estimates of PM_2.5_ concentrations during each week of pregnancy for each UGAAR participant [30].

We obtained birth weight, length, head circumference, gestational age, sex, and mode of delivery from medical records. We also collected information from medical records on stillbirths, pregnancy complications and co-morbidities [31]. Participants self-reported the occurrence and timing of spontaneous abortions.

### Postnatal data collection

Between February 2016 and January 2017, we invited all living UGAAR mother–child dyads to continue in a follow-up study of health and development in childhood. We re-enrolled dyads when the children were a median of 15.4 months of age (range: 7.7 to 28.9). Participants again provided written informed consent.

We made annual visits to participants’ homes, roughly corresponding with the child’s birthdays. During the first of these home visits we also assessed nurturing and stimulation of the child using the Home Observation Measurement of the Environment (HOME) inventory [32]. We again measured PM_2.5_ concentrations over 7 days in a subsample of participants’ homes based on availability of Dylos laser particle counters. Shortly after re-enrollment and at 6-month intervals thereafter, we asked participants to complete questionnaires about family characteristics and the child’s home environment, diet, activities, and health.

Mothers and children were invited to our study office when the children were approximately 2 and 4 years of age. At the 2-year visit, we obtained a venous whole blood sample from children for analysis of lead, mercury, and cadmium concentrations [31]. At the 2-year visit, trained assessors also administered the matrix reasoning and vocabulary subtests of the Wechsler Abbreviated Scale of Intelligence (WASI) and the Beck Depression Inventory-II (BDI) to the mothers [33].

### Assessment of behavioral outcomes

We administered the Behavior Assessment System for Children (BASC-3) to caregivers during the 2-year visits (January 2014 to December 2015) and 4-year visits (January 2016 to December 2017) in our study office. We obtained BASC-3 data for a total of 407 children including 391 children (214 intervention, 177 control) at age 2 and 388 children (205 intervention, 183 control) at age 4. The BASC-3 asks caregivers how frequently their child exhibited 139 specific behaviors or activities over the past several months. All English BASC-3 materials were translated by native Mongolian speakers. The translations were then back translated to English. We piloted the BASC-3 on Mongolian children, updated the translations, and piloted with additional children prior to finalizing the translation to assess the UGAAR children. Research staff who administered and scored the BASC-3 were blinded to intervention status.

Our primary outcomes were the four BASC-3 composite scales: externalizing problems, internalizing problems, adaptive skills, and the behavioral symptoms index (BSI). For externalizing problems, internalizing problems, and the BSI higher scores indicate more behavioral problems. For adaptive skills, lower scores indicate poorer functioning.

### Sample size

The UGAAR study was originally designed to evaluate the effects of HEPA cleaners on fetal growth, so our sample size calculations were based on term birth weight. Assuming a type I error rate of 0.05 (2-sided) and a type II error rate of 0.20, we estimated that 460 participants, in equal numbers in the treatment and control groups, were needed. We targeted a population of 540 participants assuming 18% attrition due to withdrawal and pregnancy loss.

### Data analysis

To evaluate the influence of the intervention on indoor PM_2.5_ concentrations, we compared 7-day measurements of indoor residential PM_2.5_ between intervention and control participants during pregnancy and after delivery. To account for temporal variations in outdoor PM_2.5_ concentrations and lack of independence in repeated measurements in some homes, we regressed measured PM_2.5_ concentrations on intervention status in a mixed model with random participant intercept while adjusting for month of measurement.

To our knowledge, this was the first use of the BASC-3 in Mongolia. Calculation of composite indices from BASC-3 responses requires the conversion of raw scores into sex-specific T scores based on the distribution of scores in a reference population. Since there was no Mongolian reference population with which to normalize BASC-3 scores, we transformed the UGAAR raw scores to have the same mean and variance as the US reference population (Table S1). This allowed us to then estimate sex-specific T-scores according to the BASC-3 protocol.

Our primary analysis was by intention-to-treat (ITT) and included 478 children. The complete-case analyses included 407 children whose caregivers completed the BASC-3 on at least one-time point (391 at age two and 388 children at age four). The 478 children in the ITT analysis represent the full study population except those who withdrew prior to baseline data collection (*N* = 8), known pregnancy losses and neonatal deaths (*N* = 51), and three children with conditions unrelated to air pollution that could affect behavior or our ability to reliably impute BASC-3 scores (one child with Down’s syndrome, one with cerebral palsy, and one with a hearing and speech impairment).

We used multiple imputation with chained equations (MICE) to impute outcome and covariate data for 71 children. We created 20 imputed data sets stratified by treatment group (SAS Proc MI and PROC Mianalyze). Variables included in the imputation model were those that met one of two criteria: 1) variables associated (*p* < 0.20) with the outcome that were missing for < 15% of participants, 2) variables associated (*p* < 0.05) with missingness in the outcome variable that were missing for < 15% of participants. Variables that met these criteria included maternal age at baseline, marital status at baseline, maternal smoking at baseline, maternal self-reported stress level at baseline, maternal vitamin use at baseline, maternal pre-pregnancy BMI, and living with a smoker late in pregnancy. We also included in the imputation model the outcome variables (BASC-3 T-scores at ages 2 and 4) and all exposure and adjustment variables for our primary and secondary analyses (Figure S2).

In our primary ITT analysis, we regressed outcomes on a binary intervention variable in both unadjusted models and models adjusting for preterm birth (PTB), which is defined as a birth at < 37 weeks gestation. We previously reported that the HEPA cleaner intervention was associated with a decreased risk of spontaneous abortion but an increased risk of preterm birth in this cohort [31]. We hypothesized that the intervention may have enabled fetuses who might have otherwise died in utero to be born preterm. We also evaluated the effect of the intervention in models stratified by child’s sex.

In a secondary analysis, we evaluated the associations between BASC-3 composite indices and trimester-specific and full-pregnancy averaged indoor PM_2.5_ concentrations estimated from a previously developed model of weekly concentrations [30]. The R^2^ for that model in a tenfold cross validation was 81.5% [30]. We used a directed acyclic graph to select adjustment variables in our analysis of PM_2.5_ concentrations and behavior (Figure S1). We identified four variables as potential confounders: intervention status, maternal age at baseline, family income at baseline, and living with a smoker during pregnancy. In addition, to improve precision in our estimates we also adjusted for variables that, while unlikely to be confounders, contribute to variability in parent-reported behavior and are unlikely to be on the causal pathway between PM_2.5_ and behavior: mother’s self-reported depression score on the BDI and maternal matrix reasoning and vocabulary subtest scores on the WASI. Trimester-specific models were also adjusted for PM_2.5_ concentrations in other trimesters. We did not adjust for sex because BASC T-scores were derived from sex-specific distributions for the reference population. To allow for comparisons of effect estimates from the full pregnancy and different trimesters of exposure we scaled our effect estimates to the interquartile ranges (IQR) of PM_2.5_ concentrations.

In both our primary and secondary analyses, we analyzed data collected when children were 2 years old and 4 years old separately and with the two time points combined. We used multiple linear regression for the age-specific models, and in the analysis combining 2- and 4-year data we used linear mixed models with random participant intercepts to account for repeated measurements of behavioral outcomes. We used variance inflation factors (VIF) to evaluate multicollinearity in the multiple linear regression models. All analyses were conducted using SAS 9.4 (SAS Institute Inc., Cary, NC USA).

## Results

**Fig. 1:**
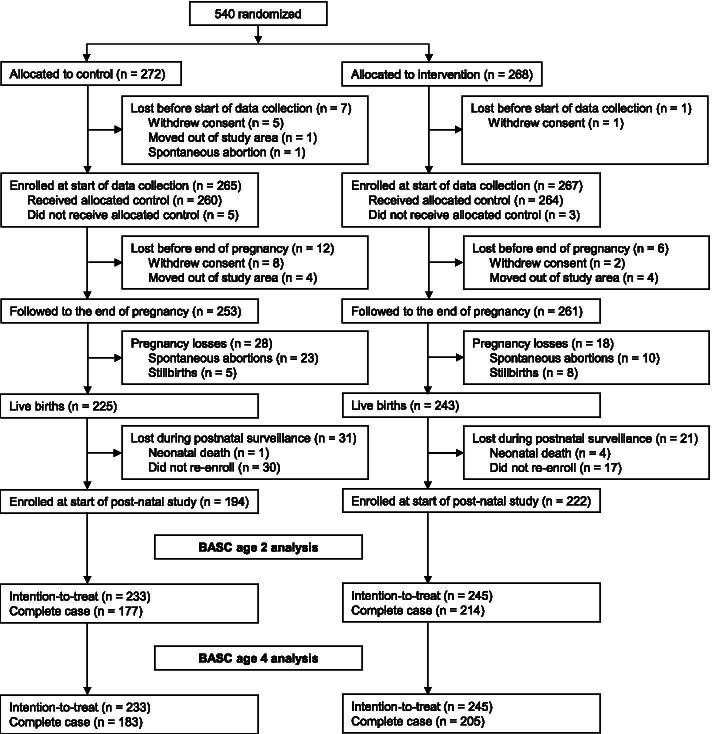
Trial profile

We recruited 540 participants (272 control and 268 intervention) from January 9, 2014 to May 1, 2015. Participants were enrolled at a median (25^th^, 75^th^ percentile) gestational age of 11 (9, 13) weeks. There were 532 participants enrolled at the start of data collection, 468 known live births, and five neonatal deaths (Fig. 1). At baseline, control and intervention participants had similar characteristics (Table 1). For example, mothers’ median (25^th^, 75^th^ percentile) ages at enrollment were 28 [25, 33] years in the control group and 29 [25, 33] years in the intervention groups. In both groups, 80% of participants reported completing university.

**Table 1:** Comparison of baseline characteristics for control and intervention participants included in the intention-to-treat analysis

Characteristic	n (%)	Control (*n* = 233)	Intervention (*n* = 245)
		Median (25^th^, 75^th^ percentile) or n (%)	Median (25^th^, 75^th^ percentile) or n (%)
Season of enrollment			
Winter (Dec, Jan, Feb)	151 (32)	77 (33)	74 (30)
Spring (Mar, Apr, May)	138 (29)	73 (31)	65 (27)
Summer (Jun, July, Aug)	56 (12)	24 (11)	32 (13)
Fall (Sep, Oct, Nov)	133 (28)	59 (25)	74 (30)
Gestational age, wk	478 (100)	10 (9, 12)	11 (9, 13)
Maternal age, yr	478 (100)	28 (25, 33)	29 (25, 33)
Monthly household income, Tugriks			
≥ 800,000	376 (79)	182 (78)	194 (79)
< 800,000	93 (19)	46 (20)	47 (19)
Not reported, n (%)	9 (2)	5 (2)	4 (2)
Maternal marital status			
Married	282 (59)	135 (58)	147 (60)
Engaged/Common-law	180 (38)	88 (38)	92 (38)
Single	10 (2)	7 (3)	3 (1)
Not reported, n (%)	6 (1)	3 (1)	3 (1)
Maternal education			
Completed university	383 (80)	187 (80)	196 (80)
Less than university	60 (13)	31 (13)	29 (12)
Not reported, n (%)	35 (7)	15 (6)	20 (8)
Lived with a smoker baseline			
No	248 (52)	120 (52)	128 (52)
Yes	218 (46)	107 (46)	111 (45)
Not reported, n (%)	12 (3)	6 (3)	6 (2)
Maternal pre-pregnancy BMI, kg/m2	449 (94)	21.7 (19.6, 23.9)	21.4 (19.7, 24.0)
Not reported, n (%)	29 (6)	21 (9)	8 (3)
Paternal age, yr	453 (95)	31 (26, 35)	30 (26, 35)
Not reported, n (%)	25 (5)	9 (4)	16 (7)
Paternal education			
Completed university	364 (76)	179 (77)	185 (76)
Less than university	85 (18)	45 (19)	40 (16)
Not reported, n (%)	29 (6)	9 (4)	20 (8)

During pregnancy, the HEPA cleaners reduced mean indoor PM_2.5_ concentrations by 29% (95% CI: 21%, 37%) from a geometric mean of 24.5 µg/m^3^ in control homes to 17.3 µg/m^3^ in intervention homes [27]. The post-natal PM_2.5_ concentrations were similar between groups (3% lower in the intervention group, 95% CI: -14%, 7%).

The characteristics of mother–child dyads in the intervention and control groups were generally similar during pregnancy and during postnatal follow-up (Table 2). The median maternal blood lead concentration late in pregnancy was 1.5 (1.2, 1.8) ug/dL in the control group and 1.4 (1.2, 1.9) ug/dL in the intervention group. As previously reported, there were more preterm births in the intervention group (10%) than in the control group (5%). In both groups, approximately 5% of mothers reported moderate to severe depression during the 2-year visit (Table 2).

**Table 2:** Comparison of pregnancy and post-natal characteristics for control and intervention participants included in the intention-to-treat analysis

Characteristic	n (%)	Control (*n* = 233)	Intervention (*n* = 245)
		Median (25^th^, 75^th^ percentile) or n (%)	Median (25^th^, 75^th^ percentile) or n (%)
Maternal blood lead concentration in late pregnancy ug/dL	375 (78)	1.5 (1.2, 1.8)	1.4 (1.2, 1.9)
Missing, n (%)	103 (22)	61 (26)	42 (17)
Type of birth			
Cesarean	174 (36)	86 (37)	88 (36)
Vaginal	285 (60)	134 (57)	151 (62)
Missing, n (%)	19 (4)	13 (6)	6 (2)
Preterm birth			
Preterm (< 37 weeks)	31 (7)	10 (4)	21 (9)
Full tern (≥ 37 weeks)	428 (89)	210 (90)	218 (88)
Missing, n (%)	19 (4)	13 (6)	6 (3)
Birth weight, grams	458 (96)	3450 (3125, 3800)	3550 (3200, 3800)
Missing, n (%)	19 (4)	13 (6)	6 (2)
Child’s sex			
Female	220 (46)	110 (47)	110 (45)
Male	239 (50)	111 (48)	128 (52)
Missing, n (%)	19 (4)	13 (5)	6 (3)
Child’s blood lead concentration at age 2, ug/dL	328 (69)	2.6 (1.9, 3.6)	2.5 (1.7, 3.5)
Missing, n (%)	150 (31)	80 (46)	70 (45)
Maternal depression level (BDI) at 2-year visit			
Mild	369 (77)	168 (72)	201 (82)
Moderate to severe	31 (6)	8 (4)	13 (5)
Missing, n (%)	88 (18)	57 (24)	31 (13)
Maternal WASI matrix reasoning raw score	387 (81)	16 (12, 19)	17 (13, 19)
Missing, n (%)	91 (19)	58 (25)	33 (13)
Maternal WASI vocabulary raw score	387 (81)	36 (32, 40)	36 (32, 40)
Missing, n (%)	91 (19)	58 (25)	33 (13)

*BDI* Beck Depression Inventory-II, *WASI* Wechsler Abbreviated Scale of Intelligence

We found no significant difference in the mean BASC-3 scores by treatment group in our ITT analysis of 478 participants (Table 3). Results were not sensitive to adjustment for PTB and results from the complete case analysis also indicated no benefits from this intervention (Table S2). Children’s behavior T scores measured 2 and 4 years of age were moderately correlated, ranging from *r* = 0.39 (adaptive skills) to 0.46 (BSI). In a stratified analysis we found no benefits of the intervention among girls or boys and no difference in effect estimates between sexes.

**Table 3:** Estimated effects of the intervention on BASC composite scores in an intention-to-treat analysis

**Composite score**	**Unadjusted**		**Adjusted for preterm birth**	
	**Change in mean T score (95%CI)**	***p*-value**	**Change in mean T score (95%CI)**	***p*-value**
**Age 2**				
Externalizing	0.21 (-1.69, 2.12)	0.83	0.21 (-1.70, 2.12)	0.83
Internalizing	-0.84 (-2.84, 1.16)	0.41	-0.84 (-2.84, 1.16)	0.41
Adaptive skills	-0.06 (-1.77, 1.64)	0.94	-0.01 (-1.73, 1.71)	0.99
Behavioral Symptoms Index	-0.28 (-1.98, 1.42)	0.75	-0.29 (-1.99, 1.40)	0.73
**Age 4**				
Externalizing	0.09 (-1.98, 2.17)	0.93	0.09 (-1.98, 2.17)	0.93
Internalizing	-0.48 (-2.51, 1.54)	0.64	-0.47 (-2.50, 1.55)	0.65
Adaptive skills	-0.29 (-2.12, 1.55)	0.76	-0.17 (-1.99, 1.66)	0.86
Behavioral Symptoms Index	-0.21 (-2.17, 1.76)	0.84	-0.20 (-2.17, 1.76)	0.84
**Ages 2 and 4 combined**^**a**^				
Externalizing problems	0.15 (-1.54, 1.85)	0.86	0.13 (-1.55, 1.82)	0.88
Internalizing problems	-0.66 (-2.36, 1.04)	0.44	-0.60 (-2.29, 1.10)	0.49
Adaptive skills	-0.18 (-1.65, 1.30)	0.82	-0.09 (-1.56, 1.38)	0.91
Behavioral symptoms index	-0.24 (-1.81, 1.32)	0.76	-0.28 (-1.84, 1.28)	0.72

^a^Age 2 and 4 BASC scores modeled together in a linear mixed effects model

**Fig. 2:**
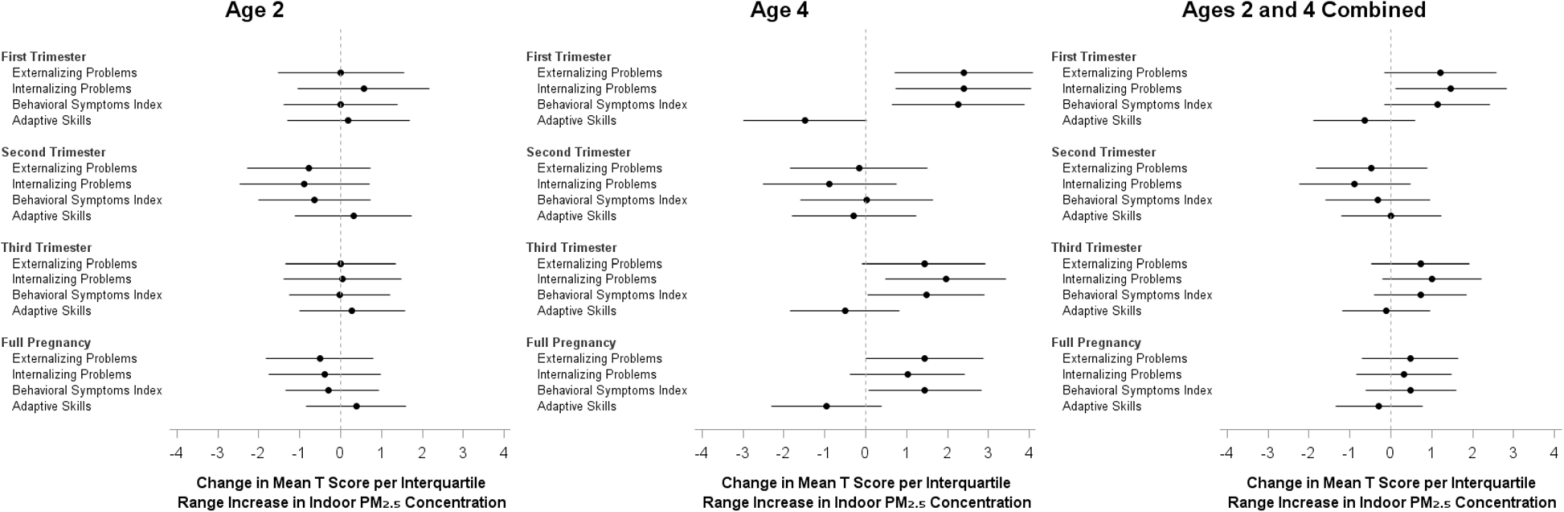
Associations between indoor PM_2.5_ concentrations during pregnancy and BASC composite scores at age 2, age 4, and combined^1^. ^1^Age 2 and 4 BASC scores modeled together in a linear mixed effects model. 1^st^ trimester IQR = 20.1 ug/m^3^, 2^nd^ trimester IQR = 21.6 ug/m^3^, 3^rd^ trimester IQR = 13.5 ug/m^3^, full pregnancy IQR = 9.63 ug/m^3^. Models adjusted for intervention status, maternal age at baseline, monthly family income at baseline, living with a smoker at any time during pregnancy, mother’s depression score at the 2-year visit, and maternal WASI matrix reasoning and vocabulary scores at the 2-year visit. Trimester-specific models were also adjusted for PM_2.5_ concentrations in other trimesters

In our secondary observational analysis we did not observe any associations between PM_2.5_ concentrations and behavior scores at age 2 (Fig. 2). We did, however, find that indoor PM_2.5_ during the first trimester of pregnancy was consistently associated with worse behavior scores at age 4 (Fig. 2). Specifically, an IQR increase (20.1 µg/m^3^) in PM_2.5_ was associated with difference of 2.4 units (95% CI: 0.7, 4.1), 2.4 units (95% CI: 0.7, 4.0), 2.3 units (95% CI: 0.7, 3.9), and -1.5 units (95% CI: -3.0, 0.0) in mean externalizing problems, internalizing problems, BSI, and adaptive skills T scores, respectively. For comparison, the estimated effect of maternal depression (moderate/severe vs. mild) on BASC scores at age 4 was 2.7 points (95% CI: -1.8, 7.1). Third trimester PM_2.5_ concentrations were associated with some behavioral indices, but effect estimates were smaller. We did not observe associations between PM_2.5_ exposure in the second trimester and any of the BASC indices (Fig. 2). Variance inflation factors were < 1.7 for all variables.

## Discussion

In this cohort of women living in a heavily polluted community, we found no evidence that reducing indoor particulate matter with HEPA air cleaners starting late in the first trimester of pregnancy improved parent-reported behavioral problems. In a secondary observational analysis, we found no associations between PM_2.5_ concentration during pregnancy and behavior at age 2. We did, however, find that first trimester PM_2.5_ concentrations were consistently associated with worse behavioral problem scores when children were 4 years old. Third trimester PM_2.5_ concentration were also associated with some behavioral indices at age 4, but effects were smaller. PM_2.5_ concentrations during the second trimester were not associated with behavior at any age. These results suggest that behavior scores in childhood may be particularly sensitive to air pollution exposure early in pregnancy, but these are exploratory findings and should be interpreted cautiously.

Brain development is a rapid and complex process and the timing of exposure to neurotoxicants may be an important determinant of adverse impacts [15, 34, 35]. Our finding of consistent associations with exposure in the first trimester is similar to a recent analysis of 4 to 6-year-old children in Mexico City [11]. In that study, which assessed behavior using an earlier version of the BASC (BASC-2), a 5 µg/m^3^ increase in outdoor PM_2.5_ concentration during the first trimester was associated with a 1.1 unit (95% CI: -0.2, 2.4) increase in mean BSI T score and a decrease of 1.5 units (95% CI: -2.6, -0.3) in mean adaptive skills T score. PM_2.5_ concentrations in other trimesters were not associated with behavior scores. In the Upstate KIDS study, a 10 µg/m^3^ increase in PM_2.5_ exposure during the first and third trimesters was associated with increases of 1.6% (95% CI: 0.1%, 3.2%) and 2.7% (95% CI: 0.6%, 4.9%), respectively, in the risk of a failed developmental screening between 8 and 36 months of age. The estimated effect of second trimester exposure to PM_2.5_ was smaller [7].

For individual children, a change in behavioral symptoms T scores of 1–3 units would generally not be problematic. At a population level, however, these impacts could be important given that 90% of the world’s population is exposed to PM_2.5_ above the World Health Organization guideline concentration [36, 37].

Our study may have been influenced by the live birth bias. Bias is possible if the intervention affects the probability of a live birth and other unmeasured exposures affect both the probability of a live birth and BASC scores. We previously reported that the air cleaner intervention was associated with a lower risk of spontaneous abortion in this cohort [31]. Thus, unmeasured exposures that increase risk of both fetal death and behavioral problems may have led us to underestimate the benefits of this intervention [38, 39].

This study had several limitations. We may have been underpowered for this outcome as the sample size calculation was based on term birth weight, the original outcome of this trial. Staff who administered and scored the BASC-3 were blinded to the participants’ group assignment, but the participants were not blinded. The lack of blinding could also partly explain the greater loss of control participants. This may have introduced some selection bias, which we tried to address through an ITT analysis using multiple imputation. To our knowledge, this was the first use of the BASC-3 in Mongolia. We translated the BASC-3 using an iterative process that included back-translation and extensive pilot testing in Mongolian children before collecting data from UGAAR participants. However, even careful translation may not account for important cultural differences in the way behaviors are described and interpreted [40]. Behaviors may be accepted differently in different settings, so use of an instrument designed for North American children may not have fully captured behavior problems in this cohort of Mongolian children. This would likely have introduced non-differential errors leading to underestimated effects. In addition, because there was no Mongolian reference population, we scaled the raw scores to match those of the US reference population before calculating scaled scores and composite indices. While this may have introduced some error in the BASC-3 scores, these errors were also likely non-differential. Although we demonstrated that the intervention reduced PM_2.5_ in participants’ homes, we did not measure personal exposure to PM_2.5_ and the impacts of this residential intervention would be attenuated by exposure encountered outside of the home. In our secondary exploratory analysis, we ran numerous models involving many combinations of exposure periods, behavioral indices, and children’s ages.

## Conclusions

In this randomized controlled trial, we found no benefit of reducing indoor air pollution during pregnancy on parent-reported behaviors in children at 2–4 years of age. In secondary analyses, however, we found that first trimester residential PM_2.5_ concentrations were associated with worse behavior scores at 4 years of age, suggesting that it may be necessary to intervene early in pregnancy to protect children. However, these exploratory findings should be interpreted cautiously and require replication.

## Supplementary Information

**Additional file 1: Table S1.** Comparison of BASC score distributions between the UGAAR population and the reference population. **Table S2.** Effects of the air cleaner intervention on BASC composite scores at ages 2 and 4 estimated from a mixed effects model among complete cases. **Table S3.** Comparison of baseline characteristics for participants who did and did not complete the BASC. **Figure S1.** Directed acyclic graph used to identify adjustment variables in models of the association between PM_2.5_ and BASC scores. **Figure S2.** Data analysis scheme for intention-to-treat analyses of combined 2-year and 4-year BASC scores using linear mixed effects models.

## Data Availability

The datasets used and/or analyzed during the current study are available from the corresponding author on reasonable request.

## Abbreviations

BASC-3: Behavior Assessment System for Children, 3^rd^ Edition
BDI: Beck Depression Inventory-II
BSI: Behavioral skills index
HEPA: High efficiency particulate air/arresting
HOME: Home Observation Measurement of the Environment
ITT: Intention-to-treat
IQR: Interquartile range
MICE: Multiple imputation with chained equations
PSS4: 4-Question perceived stress scale
RCT: Randomized controlled trial
UGAAR: Ulaanbaatar Gestation and Air Pollution Research
VIF: Variance inflation factors
WASI: Wechsler Abbreviated Scale of Intelligence
