# Supplementary material for: Portable HEPA filter air cleaner use during pregnancy and children’s behavior problem scores: a secondary analysis of the UGAAR randomized controlled trial
Source: Environ Health. 2021 Jul 5;20:78. doi: 10.1186/s12940-021-00763-6 (PMC8258951; doi:10.1186/s12940-021-00763-6)

**Online Supplement**

**Table S1.** Comparison of BASC score distributions between the UGAAR population and the reference population.

| **Domain** | **UGAAR raw scores**  **Mean (SD)** | | **Reference raw scores**  **Mean (SD)** | |
| --- | --- | --- | --- | --- |
|  | **Boys (n=201)** | **Girls (n=190)** | **Boys** | **Girls** |
| Hyperactivity | 12.26 (4.10) | 12.14 (4.72) | 11.72 (5.20) | 10.43 (5.23) |
| Aggression | 4.23 (2.86) | 4.07 (3.01) | 3.61 (2.93) | 3.32 (3.06) |
| Anxiety | 4.80 (3.54) | 5.04 (4.09) | 4.62 (4.37) | 4.45 (4.20) |
| Depression | 7.72 (3.06) | 7.88 (4.01) | 7.89 (4.12) | 7.64 (4.43) |
| Somatization | 7.16 (3.66) | 6.98 (3.90) | 5.27 (3.66) | 5.71 (4.86) |
| Attention Problems | 8.94 (3.11) | 8.61 (3.03) | 8.11 (3.69) | 7.38 (3.35) |
| Atypicality | 6.26 (3.44) | 6.59 (3.86) | 3.76 (3.56) | 3.20 (3.93) |
| Withdrawal | 10.39 (4.41) | 10.34 (4.59) | 9.69 (4.85) | 10.43 (5.13) |
| Adaptability | 14.75 (3.86) | 14.79 (4.30) | 16.15 (4.84) | 16.60 (4.58) |
| Social Skills | 10.74 (4.66) | 11.97 (5.61) | 13.91 (5.92) | 15.99 (5.42) |
| Activities of Daily Living | 8.33 (4.32) | 9.39 (4.25) | 8.82 (4.33) | 10.13 (3.84) |
| Functional Communication | 11.13 (3.96) | 12.68 (4.60) | 16.84 (6.77) | 18.40 (6.33) |

**Table S2.** Effects of the air cleaner intervention on BASC composite scores at ages 2 and 4 estimated from a mixed effects model among complete cases.

| **Composite score** | **Unadjusted model** | | | **Adjusted for preterm birth** | | |
| --- | --- | --- | --- | --- | --- | --- |
|  | **N** | **Change in mean T score (95%CI)** | **p-value** | **N** | **Change in mean T score (95%CI)** | **p-value** |
| Externalizing | 407 | 0.34 (-1.27, 1.94) | 0.68 | 406 | 0.33 (-1.28, 1.94) | 0.69 |
| Internalizing | 407 | -0.59 (-2.21, 1.02) | 0.47 | 406 | -0.57 (-2.19, 1.05) | 0.49 |
| Adaptive | 407 | -0.07 (-1.49, 1.35) | 0.93 | 406 | 0.03 (-1.39, 1.45) | 0.96 |
| Behavior Symptom Index | 407 | -0.07 (-1.58, 1.43) | 0.92 | 406 | -0.12 (-1.63, 1.39) | 0.88 |

**Table S3.** Comparison of baseline characteristics for participants who did and did not complete the BASC.

| **Baseline characteristic** | **Completed BASC-3**  Median (25^th^, 75^th^ percentile) or n (%) | | **Did not complete BASC-3**  Median (25^th^, 75^th^ percentile) or n (%) | p-value |
| --- | --- | --- | --- | --- |
| Season of enrollment |  |  | |  |
| Winter (Dec, Jan, Feb) | 124 (31) | 27 (38) | | 0.25 |
| Spring (Mar, Apr, May) | 115 (28) | 23 (32) | |  |
| Summer (Jun, July, Aug) | 54 (13) | 2 (3) | |  |
| Fall (Sep, Oct, Nov) | 113 (28) | 20 (27) | |  |
| Gestational age, wk | 11 (8, 13) | 11 (9, 13) | | 0.11 |
| Maternal age, yr | 29 (25, 33) | 27 (24.5, 32) | | 0.17 |
| Monthly household income, Tugriks |  |  | |  |
| < 800,000 | 79 (20) | 14 (19) | |  |
| ≥ 800,000 | 322 (79) | 54 (75) | | 0.87 |
| Not reported, n (%) | 5 (1) | 4 (6) | |  |
| Maternal marital status |  |  | |  |
| Married/ Common-law | 339 (83) | 56 (78) | | 0.24 |
| Single/engaged | 67 (17) | 16 (22) | |  |
| Not reported, n (%) |  |  | |  |
| Maternal education |  |  | |  |
| Less than university | 50 (12) | 10 (14) | |  |
| Completed university | 328 (81) | 55 (76) | | 0.64 |
| Not reported, n (%) | 28 (7) | 7 (10) | |  |
| Lived with a smoker during pregnancy |  |  | |  |
| No | 211 (52) | 37 (51) | |  |
| Yes | 185 (46) | 33 (46) | | 0.95 |
| Not reported, n (%) | 10 (2) | 2 (3) | |  |
| Maternal pre-pregnancy BMI, kg/m2 | 21.6 (19.6, 24.2) | 21.3 (19.6, 23.1) | | 0.37 |
| Not reported, n (%) | 28 (7) | 1 (1) | |  |
| Paternal age, yr | 31 (26, 35) | 29 (25, 34) | | 0.25 |
| Not reported, n (%) | 24 (6) | 1 (1) | |  |
| Paternal education |  |  | |  |
| Less than university | 71 (17) | 14 (19) | |  |
| Completed university | 308 (76) | 56 (78) | | 0.80 |
| Not reported, n (%) | 27 (7) | 2 (3) | |  |

**Figure S1.** Directed acyclic graph used to identify adjustment variables in models of the association between PM_2.5_ and BASC scores.


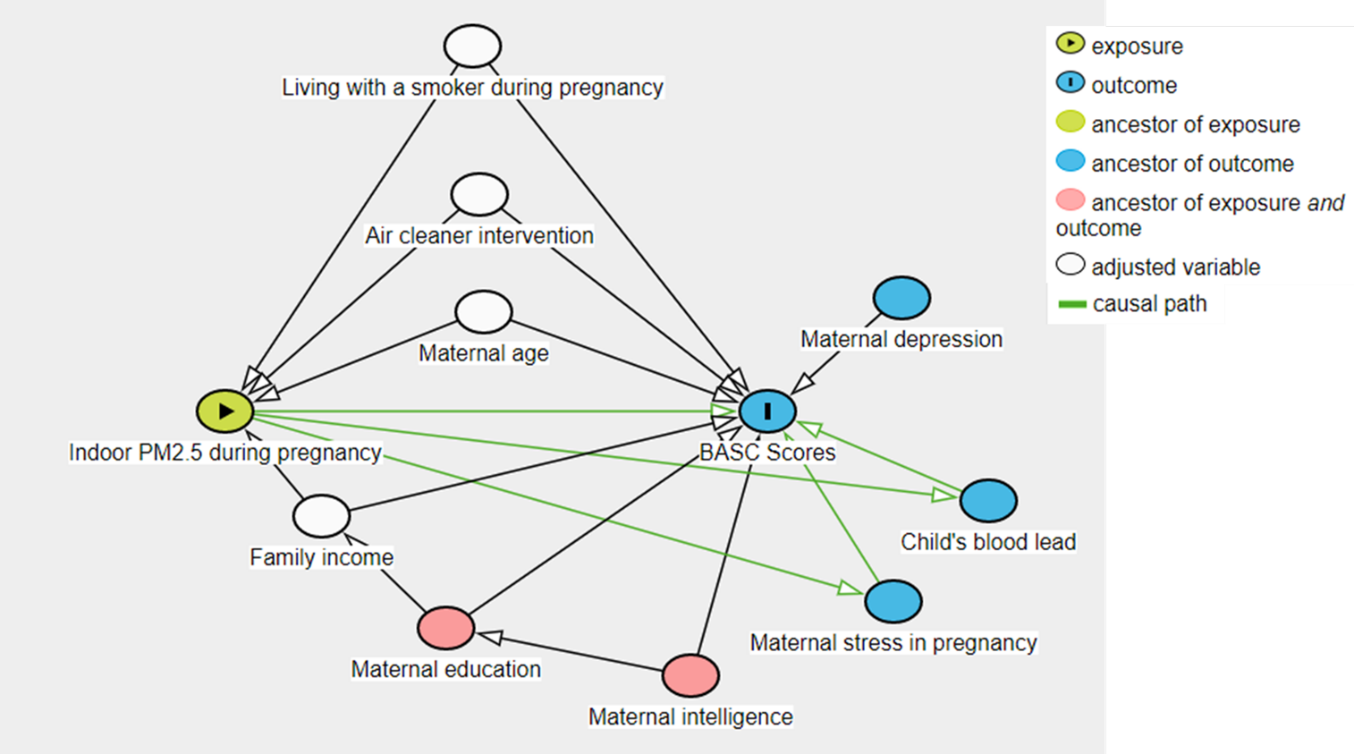


**Figure S2.** Data analysis scheme for intention-to-treat analyses of combined 2-year and 4-year BASC scores using linear mixed effects models.


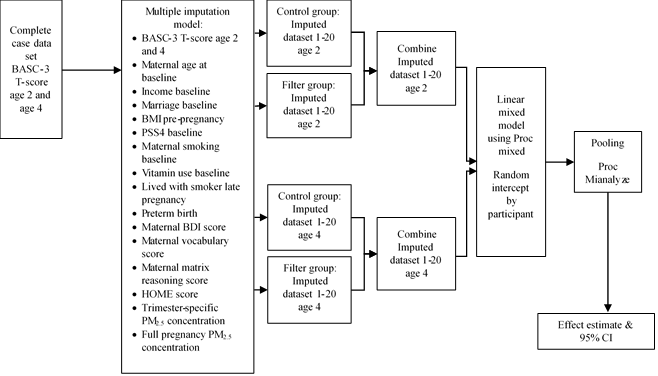

Supplement: Supplementary file 1 — Additional file 1: Table S1. Comparison of BASC score distributions between the UGAAR population and the reference population. Table S2. Effects of the air cleaner intervention on BASC composite scores at ages 2 and 4 estimated from a mixed effects model among complete cases. Table S3. Comparison of baseline characteristics for participants who did and did not complete the BASC. Figure S1. Directed acyclic graph used to identify adjustment variables in models of the association between PM2.5 and BASC scores. Figure S2. Data analysis scheme for intention-to-treat analyses of combined 2-year and 4-year BASC scores using linear mixed effects models. [file 12940_2021_763_MOESM1_ESM.docx]
